# Innate host defense lysozyme may help control *Staphylococcus aureus* in atopic dermatitis

**DOI:** 10.1128/spectrum.01585-26

**Published:** 2026-08-11

**Authors:** Patrick M. Schlievert, Samuel H. Kilgore, Evgeny Berdyshev, Michael Nodzenski, Gloria David, Takeshi Yoshida, Lisa A. Beck, Donald Y. M. Leung

**Affiliations:** 1 Department of Microbiology and Immunology, Carver College of Medicine, University of Iowa 4083, Iowa City, Iowa, USA; 2 Department of Medicine, National Jewish Health 2930, Denver, Colorado, USA; 3 Rho, Inc., Durham, North Carolina, USA; 4 Department of Dermatology, University of Rochester Medical Center 6923, Rochester, New York, USA; 5 Department of Pediatrics, National Jewish Health 2930, Denver, Colorado, USA; Emory University School of Medicine, Atlanta, Georgia, USA

**Keywords:** lysozyme, *Staphylococcus aureus*, atopic dermatitis

## Abstract

Atopic dermatitis (AD) is a chronic pruritic skin condition characterized by lesions associated with colonization and infection by *Staphylococcus aureus*. There is growing evidence that *S. aureus* contributes to symptoms and persistence of AD through production of cytotoxins, superantigens, and proteases. When AD is treated with the interleukin-4 receptor blocking antibody, dupilumab, *S. aureus* colonization declines rapidly. Here, we examined the possible involvement of the host lysozyme-mediated innate immune response in helping to control *S. aureus* burden in AD patients. *S. aureus* is not killed by lysozyme, but the molecule inhibits *S. aureus* exotoxin and exoenzyme (exoprotein) production. Reduced *S. aureus* abundance during dupilumab treatment correlated positively with reduced lysozyme levels. *S. aureus* colonization and lysozyme levels were not altered in placebo-treated participants until they entered the open-label phase of the study (non-blinded phase) when all participants were treated with dupilumab. During that phase, the *S. aureus* and lysozyme levels declined. These findings suggest that AD patients may respond to cutaneous *S. aureus* colonization by upregulating lysozyme production.

**IMPORTANCE:**

*Staphylococcus aureus* is increasingly viewed as an important contributor to atopic dermatitis (AD) persistence. The host responds to AD by producing cationic peptides, including lysozyme. Lysozyme cannot kill *S. aureus*, but the innate defense molecule downregulates exotoxin and exoenzyme production, limiting S. aureus-induced inflammation. Clinical improvement of AD likely requires balancing the inflammatory cascades induced by *S. aureus* versus host innate immunity.

## INTRODUCTION

Atopic dermatitis (AD) is a chronic pruritic inflammatory skin condition characterized by skin barrier dysfunction, with significant trans-epidermal water loss (1, 2). AD skin lesions demonstrate infiltration of T lymphocytes and eosinophils, and increased cutaneous expression of T helper-2 cell cytokines, including interleukin (IL)−4 and IL-13 (reviewed in reference [3]). The skin of all AD patients is colonized or infected with *Staphylococcus* aureus, all of which produce superantigens (toxic shock syndrome toxin-1, staphylococcal enterotoxins, and staphylococcal enterotoxin-like molecules) and cytotoxin (hemolysin) exotoxins (1, 4).

There are multiple clonal groups of *S. aureus*, as defined by the Centers for Disease Control and Prevention (CDC) based on pulsed-field gel electrophoresis, with a USA designation (5), and by other strategies, including multi-locus sequence typing (MLST), with a Clonal Cluster (CC) designation (6). These methods refer to common clonal groups, such as USA100 (CC5), USA200 (CC30), USA300 (CC8), and USA400 (CC1). USA200 strains, most of which produce the superantigen toxic shock syndrome toxin-1 (TSST-1), caused the vaginal menstrual TSS epidemic (7), cause highly fatal post-influenza toxic shock syndrome (TSS) with associated tracheitis (8), and are highly associated with bullous pemphigoid (9) and eczema herpeticum in children (10). AD cases associated with TSST-1 in general are more severe than other cases (11).

Intact, healthy skin provides a critical innate immune mechanism to prevent infections by many bacteria. This is accomplished through its barrier function, antimicrobial lipids, and cationic antimicrobial peptides. Many studies have shown that cationic defensin molecules kill *S. aureus*, but only at relatively high (microgram per milliliter) amounts (12, 13). However, at nearly six log_10_ lower concentrations, defensin peptides inhibit exotoxin production by *S. aureus* (12); which are required for colonization and infection. Another cationic antimicrobial peptide found on skin and mucous membrane surfaces of humans is lysozyme (14, 15). Lysozyme is approximately 14,000 molecular weight with an isoelectric point of 11.5. Although lysozyme is effective in lysing many types of bacteria, most notably *Micrococcus lysodeikticus*, lysozyme is ineffective at killing *S. aureus*, even at very high concentrations (16). However, at biologically relevant concentrations, lysozyme inhibits exotoxin production by *S. aureus*, functioning through cationic effects on staphylococcal two-component sensing systems, such as staphylococcal respiratory response (SrrAB) (16).

*S. aureus* has 16 two-component systems. These systems provide the major mechanisms for staphylococcal interaction with the external environment. Some of the two-component systems are required for bacterial survival (for example WalKR), and some are required for exotoxin production (for example SrrAB, AgrAC, and SaeRS) (17–22). From these prior studies, two-component systems were shown to be important targets for antimicrobial and anti-exotoxin activity of highly positively charged (cationic) molecules.

Thus, lysozyme may contribute to reduction in *S. aureus*-induced skin inflammation in AD through its ability to interfere with the production of exotoxins and exoenzymes (exoproteins) by functioning at least through the two-component system SrrAB, despite having limited bactericidal activity against *S. aureus* (16); it is possible that lysozyme interferes with other two-component systems as well as SrrAB, but this has not been tested. SrrAB is an oxygen-sensing two-component system, and studies have shown that exotoxin and exoenzyme production by *S. aureus* depends on the presence of at least 3% oxygen (17, 18, 23, 24). Studies have shown that lysozyme-deficient model systems exhibit increased susceptibility to infections due to pathogens, such as *Klebsiella pneumoniae* in a mouse knockout pneumonia model, and macrophages in a *S. aureus* in a lysozyme-suppressed model (25, 26). One study suggested that lysozyme production is upregulated in lesional AD skin, suggesting its importance in innate defense (27).

The current study was undertaken to evaluate lysozyme amounts in a double-blinded study of AD patients who were treated with the monoclonal antibody dupilumab (blocks the signaling of IL-4 and IL-13 by binding to the IL-4R alpha subunit) versus placebo.

The data show that treatment with dupilumab leads to rapid and significant reductions in *S. aureus*. Like in the study cited above on upregulation of lysozyme synthesis in lesional skin of AD patients (27), lysozyme mRNA amounts in patients treated with dupilumab are initially increased (3–7 days) and then parallel the reductions in *S. aureus*.

## RESULTS

Our prior studies have shown that 100% of AD patients are colonized or infected with *S. aureus* (1). The quantity of culturable organisms in lesional and non-lesional skin of moderate to severe AD ranges between 10^3^ and 10^5^/cm^2^ (1). *S. aureus* abundance (by CFUs and PCR quantification [rCFUs]) decreased rapidly by 1–2 logs_10_ for lesional skin and 0.5–1 log_10_ for non-lesional skin with treatment of patients with dupilumab for 42 days, compared with not decreasing for placebo controls (1).

Total cytotoxin (α, β, γ, δ, ε, and phenol-soluble modulins) showed similar trends to the colony-forming units (CFUs)/mL. Total cytotoxin on lesional skin declined by nearly two log_10_ (to undetectable; 10^−4^ µg/cm^2^) and one log_10_ for non-lesional skin (to undetectable; 10^−4^ µg/cm^2^) (1) when comparing day 42 of the study to baseline. Placebo controls remained unchanged over the same time period.

Human lysozyme is easily quantified by lysis of killed *Micrococcus lysodeikticus*. Briefly, *M. lysodeikticus* is immobilized in agarose on microscope slides. After solidifying, wells are punched into the agarose, and skin swab samples are added to each well. Control wells contain dilutions of purified lysozyme of known concentrations. All slides are placed in humidified chambers and incubated at 37°C overnight. Subsequently, area squared of *M. lysodeikticus* lysis is measured and compared with the control lysozyme wells.

**Fig 1:**
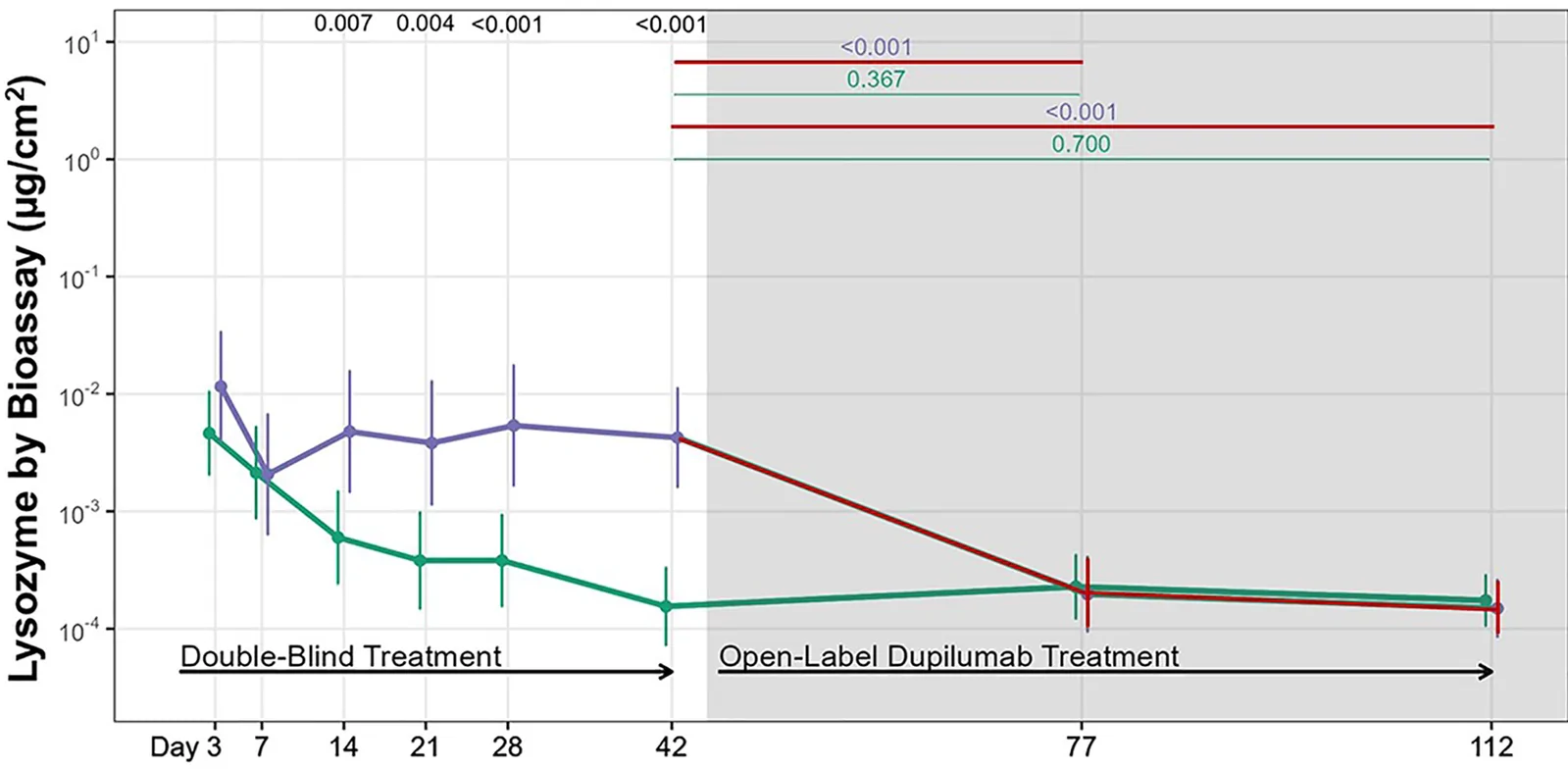
Lysozyme quantification from AD lesional skin treated with dupilumab (green line) or placebo treatment (purple line). Red line for placebo participants who received dupilumab in the open-label phase. The values plotted are estimated least-square geometric means with 95% confidence intervals. The first 42 days were double-blinded data, and thereafter, all patients (open-label) were treated with dupilumab. Lysozyme was quantified by lysis of *Micrococcus lysodeikticus* compared with lysis with known amounts of lysozyme. *P* values are listed along the top.

**Fig 2:**
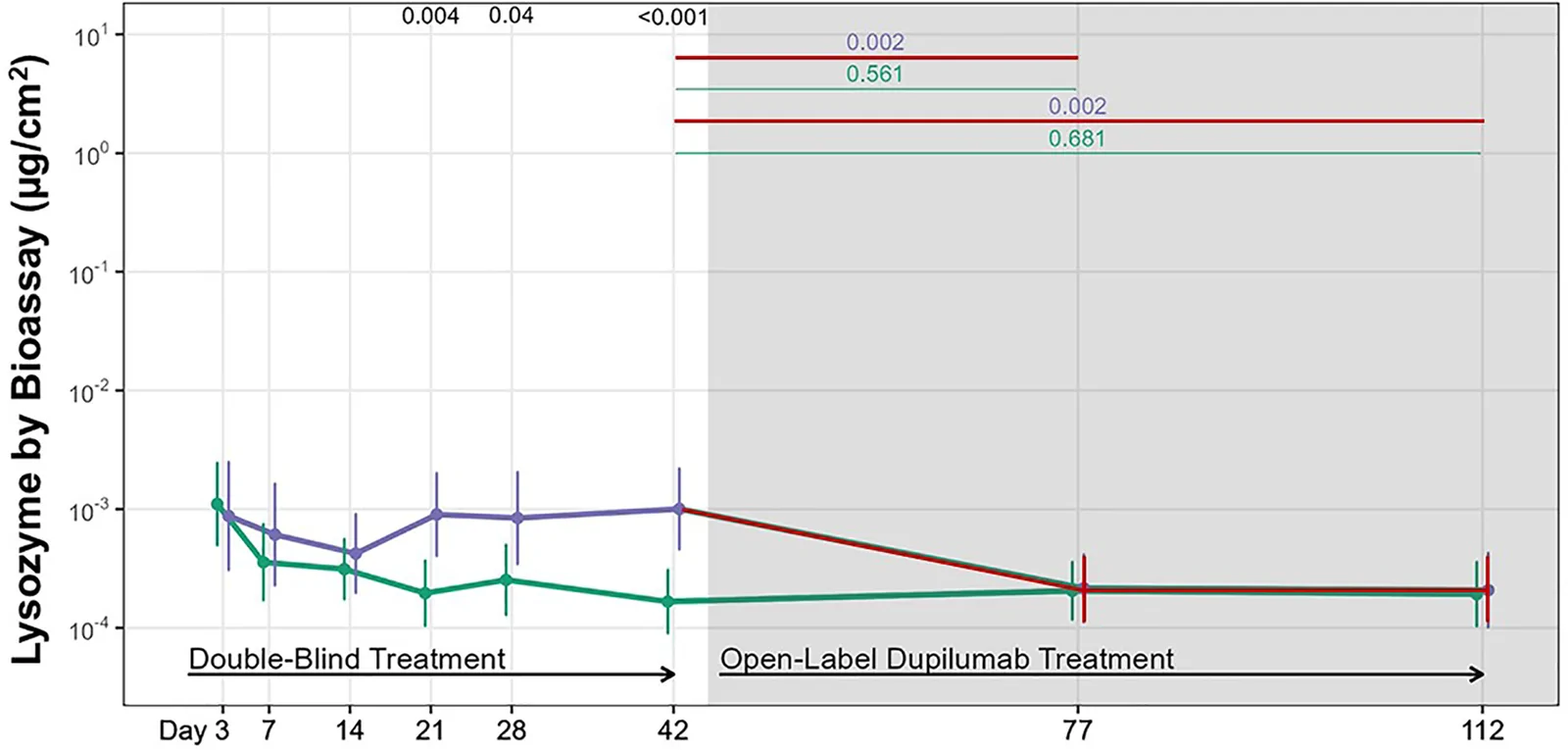
Lysozyme quantification from AD non-lesional skin treated with dupilumab (green line) or placebo treatment (purple line). The red line for placebo participants who received dupilumab in the open-label phase. The values plotted are estimated least-square geometric means with 95% confidence intervals. The first 42 days were double-blinded data, and thereafter, all patients (open-label) were treated with dupilumab. Lysozyme was quantified by lysis of *Micrococcus lysodeikticus* compared with lysis with known amounts of lysozyme. *P* values are listed along the top.

We used this assay to examine biologically active lysozyme amounts in skin swab samples collected from dupilumab-treated participants and placebo controls (lesional skin Fig. 1; non-lesional skin Fig. 2) over the same time period as *S. aureus* CFUs/cm^2^ and total cytotoxin/cm^2^. Lysozyme amounts from lesional skin were approximately 10^−2^ µg/cm^2^ initially for both dupilumab and placebo participants (Fig. 1). By days 14 to 42, lysozyme amounts from the dupilumab-treated participants had declined significantly compared with the placebo controls, which remained elevated near 10^−2^ µg/cm^2^ (*P* = 0.007 and *P* < 0.001, respectively, for comparison between groups at days 14 and 42). These data for both dupilumab and placebo-treated participants showed similar trends to the data for *S. aureus* CFUs/cm^2^ and total cytotoxin/cm^2^ over the same time period. In the open-label period, when all participants were treated with dupilumab, lysozyme remained low in the dupilumab-treated group; however, lysozyme declined rapidly in the placebo control group, again showing similar trends as *S. aureus* CFUs/cm^2^ and total cytotoxin/cm^2^.

The same type of lysozyme data were generated with non-lesional skin. We know that non-lesional skin is altered, but the CFUs of *S. aureus* and total cytotoxin amounts are not as high as in lesional skin (1). The data from non-lesional sites also showed significant reductions in lysozyme with dupilumab treatment (Fig. 2) compared with no reduction in the placebo group (*P* = 0.004 and *P* = 0.04 for days 21 and 28, respectively, and *P* < 0.001 for day 42), until the open-label period where all participants were placed on dupilumab. In the open-label period (days 77 and 112), both the dupilumab and placebo groups had lysozyme near or at background.

## DISCUSSION

AD is a chronic skin condition, with symptoms and persistence of skin lesions, with contributions from *S. aureus* colonization or infection, triggered by immune dysregulation, and barrier defects, including filaggrin defects (1, 28–30). The harmful *S. aureus* effects depend on exotoxin and exoenzyme (collectively exoproteins) production (1, 31, 32).

In response to the presence of *S. aureus* and its virulence factors in lesions, the host would be expected to mount an inflammatory response, characterized in part by the presence of antimicrobial defensins and lysozyme exerting their actions through cationic properties (1, 12, 16, 33). As we have observed, the major actions of defensins and lysozyme against *S. aureus* appear to be to prevent exoprotein production, rather than killing of the organisms. The direct targets of these cationic peptides/proteins appear to be two-component systems required for exoprotein production (12, 16).

In a prior study, we showed that treatment of AD patients with dupilumab leads to significant reduction in AD symptoms (e.g., itch), improvement in skin lesions, which is observed shortly after the reductions in *S. aureus* and its virulence factor production (1, 34). In addition, transcriptomic analysis of lesional skin biopsies 7 days after dupilumab treatment demonstrated upregulation of lysozyme transcription (1). In the current study, we showed that active, demonstrable lysozyme protein was present in the lesional and non-lesional skin of AD patients early in both dupilumab and placebo groups. Bioactive lysozyme declined as the AD symptoms abated with dupilumab treatment and correlated positively with reductions of *S. aureus* numbers. These studies suggest that the host lysozyme presence was occurring in response to *S. aureus*. As the skin lesions improved and *S. aureus* numbers declined, the host no longer required the bioactive lysozyme response to help control the infection, and the amounts of lysozyme declined.

However, in the placebo-treated patients, bioactive lysozyme levels remained high until the open-label phase of the study, where all placebo-assigned participants received dupilumab, wherein *S. aureus* numbers declined, and lysozyme levels also declined.

Collectively, these data indicate that the host attempts to use bioactive lysozyme as at least one of the innate defense mechanisms to help control *S. aureus*, but lysozyme alone cannot remove *S. aureus*. It is hypothesized that the numbers of *S. aureus* present might have been higher in skin without the presence of lysozyme, but studies have not been done to verify this hypothesis. Additional molecules, such as other cationic peptides/proteins and normalization of the AD skin affected by inflammation/alterations in skin lipid composition, are important in the removal of *S. aureus*. It does appear that lysozyme reduction may predict improvement in AD skin. Future studies should assess more in-depth whether or not this is the case.

In summary, we have shown that bioactive lysozyme protein amounts were elevated in lesional and non-lesional skin of AD patients who have high abundance of *S. aureus*, and that lysozyme levels decline as both *S. aureus* and its virulence factors are markedly reduced with dupilumab treatment. Lysozyme is likely to help in control of *S. aureus* in the lesional and non-lesional skin of AD patients.

## MATERIALS AND METHODS

### Atopic dermatitis (AD) patients

The patient population evaluated in this study was those studied previously in the Atopic Dermatitis Research Network (ADRN)−09 clinical trial, that studied the efficacy of dupilumab in adults with moderate to severe AD (ClinicalTrials.gov, NCT03389893) (1). These patients (*n* = 71) all had *S. aureus* isolated from the skin and were randomized 2:1 to receive either dupilumab or placebo for the first 6 weeks (days 1 to 42) and then all subjects entered the open-label phase where they all received dupilumab for an additional 10 weeks (days 42 to 112). The demographics of the population are shown in Table 1.

**TABLE 1:** Demographics and baseline characteristics of the participant population with atopic dermatitis

Characteristic	Dupilumab treated *N* = 45	Placebo *N* = 26
Age (years) at screening	36.6 ± 15.6^*a*^	37.3 ± 15.6
Race		
White	21	13
Asian	7	7
Black or African American	8	3
American Indian or Alaska Native	1	0
>1 race	5	1
Unknown or not reported	3	2
Body mass index (kg/m^2^)	27.5 ± 5.7	25.8 ± 4.9
EASI^*b*^ score at baseline		
<21.1	16	10
≥21.1	29	16
SCORAD^*c*^ (0-103)	62.1 ± 11.7	59.9 ± 10.9

^*a*^ Mean ± standard deviation.

^*b*^ EASI (eczema area and severity index) indicates the severity and extent of AD, evaluating four signs: erythema, edema/papulation, excoriation, and lichenification. A score of ≥21.1 = severe atopic dermatitis.

^*c*^ SCORAD (SCORing Atopic Dermatitis) is another measure of AD severity. A score of >50 is considered severe.

### Bacteriology

Skin *S. aureus* previously was assessed by both viable plate counts on mannitol salt agar from skin swabs, and by a second method, quantitative polymerase chain reaction for the specific *S. aureus femA*, a gene required for cell wall synthesis (1).

Total staphylococcal cytotoxins (α, β, γ, δ, ε, and phenol soluble modulins [PSMs]) previously were measured by a bioassay dependent on lysis of erythrocytes (1, 16).

### Lysozyme

Human lysozyme was quantified by lysis of killed *Micrococcus lysodeikticus.* Briefly, *M. lysodeikticus* (50 mg dried powder/10 mL) was immobilized in 0.85% agarose in 0.005 M NaPO_4_, pH 7.2, 0.15 M NaCl) on microscope slides (4 mL/slide). After solidifying, 4-mm wells were punched into the agarose, and 5 μL of skin swab samples (from 2 × 2 cm surface area in 2 mL of transport medium) were added to each well. Control wells contained 5 μL of dilutions of purified lysozyme of known concentrations (20, 2, 0.2, 0.02, 0.002, and 0 μg/well). All slides were placed in humidified chambers and incubated at 37°C, 5% CO_2_, overnight. Subsequently, area squared of *M. lysodeikticus* lysis was measured and compared with the control lysozyme wells.

### Statistics

We used generalized least squares fit by restricted maximum likelihood (REML) to model post-baseline, log_10_-transformed lysozyme levels, fitting separate models for lesional and non-lesional skin. For both the lesional and non-lesional models, we included treatment (dupilumab vs placebo), visit (day), and a treatment-by-visit interaction term as predictors. We did not assume a linear effect of time and instead treated visit as a categorical variable. We further adjusted for randomization-stratification variables (clinical site, baseline EASI score [≥21.1 vs <21.1]) and baseline (day 0) lysozyme level. To account for correlation of repeated within-subject lysozyme measurements, we assumed an unstructured variance-covariance matrix.

We carried out Wald tests based on the fitted model coefficients (with Kenward-Roger degrees of freedom) to assess the null hypothesis that there was no treatment effect of dupilumab relative to placebo on the mean log10 lysozyme level at each post-baseline timepoint in the blinded treatment period (through day 42). Because all participants in the open-label extension (from day 42 to 112) received dupilumab, we did not compare randomization groups during this period. Instead, we carried out Wald tests separately within each randomized group to compare lysozyme levels at days 77 and 112 to day 42. We considered all analyses to be exploratory and did not adjust *P* values for multiple tests.

To visualize results, we computed least-square (marginal) means for each treatment group and visit, assuming covariate margins equal to those observed in the study. We exponentiated (using base 10) the estimated least square means and associated 95% CI bounds to obtain geometric means and corresponding confidence intervals and then plotted these values.

We performed all statistical analyses using SAS software, version 9.4 (SAS Institute, Inc., Cary, NC, USA), and generated all figures using the ggplot2 package, version 4.0.2, in R software, version 4.4.1. Other statistical details were listed in a previous publication (1).

## Data Availability

Data are available upon request.
